# Talking to chromatin: post-translational modulation of polycomb group function

**DOI:** 10.1186/1756-8935-2-10

**Published:** 2009-09-01

**Authors:** Hanneke EC Niessen, Jeroen A Demmers, Jan Willem Voncken

**Affiliations:** 1 Molecular Genetics, GROW School for Oncology and Developmental Biology, Maastricht University, Maastricht, The Netherlands; 2 Proteomics Center, Erasmus MC University Medical Center, Rotterdam, The Netherlands

## Abstract

Polycomb Group proteins are important epigenetic regulators of gene expression. Epigenetic control by polycomb Group proteins involves intrinsic as well as associated enzymatic activities. Polycomb target genes change with cellular context, lineage commitment and differentiation status, revealing dynamic regulation of polycomb function. It is currently unclear how this dynamic modulation is controlled and how signaling affects polycomb-mediated epigenetic processes at the molecular level. Experimental evidence on regulation of polycomb function by post-translational mechanisms is steadily emerging: Polycomb Group proteins are targeted for ubiquitylation, sumoylation and phosphorylation. In addition, specific Polycomb Group proteins modify other (chromatin) associated proteins via similar post-translational modifications. Such modifications affect protein function by affecting protein stability, protein-protein interactions and enzymatic activities. Here, we review current insights in covalent modification of Polycomb Group proteins in the context of protein function and present a tentative view of integrated signaling to chromatin in the context of phosphorylation. Clearly, the available literature reveals just the tip of the iceberg, and exact molecular mechanisms in, and the biological relevance of post-translational regulation of polycomb function await further elucidation. Our understanding of causes and consequences of post-translational modification of polycomb proteins will gain significantly from *in vivo *validation experiments. Impaired polycomb function has important repercussions for stem cell function, development and disease. Ultimately, increased understanding of signaling to chromatin and the mechanisms involved in epigenetic remodeling will contribute to the development of therapeutic interventions in cell fate decisions in development and disease.

## Introduction

**Figure 1:**
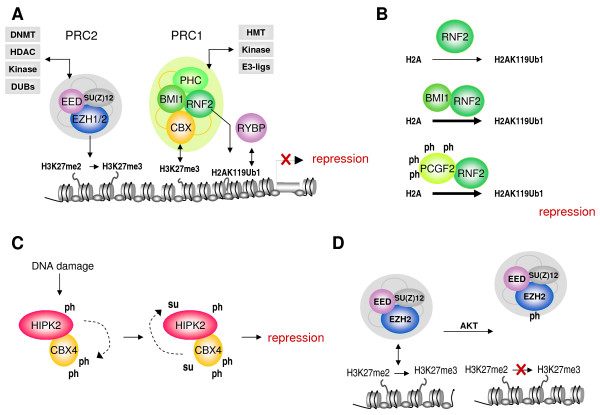
**Examples of post-translational modulation of Polycomb group and associated proteins**. **(a) **Simplified model of Polycomb Group (PcG)-mediated repression. The histone methyltransferase EZH2 trimethylates histone H3 at lysine 27, this mark is recognized by chromobox homolog (CBX) proteins via the chromodomain. RNF2/RING1 homologs are E3 ubiquitin ligases for H2A; RYBP binds H2AK119Ub1. Combined, these activities induce/maintain transcriptional repression. Gray boxes depict Polycomb Repressive Complex (PRC)-associated, epigenetically relevant enzymatic activities. **(b) **RNF2 E3 ligase activity is significantly enhanced in the presence of BMI1 or phosphorylated PCGF2. **(c) **DNA damage-induced phosphorylation of HIPK2 leads to phosphorylation of CBX4. Phosphorylation of CBX4 at T495 in turn enhances the HIPK2 sumoylation. **(d) **AKT-induced phosphorylation of EZH2 on S21 impairs its binding to histone H3, thereby inhibiting H3K27 trimethylation. me = methylation, ph = phosphorylation, su = sumoylation; ub = ubiquitylation.

Polycomb group (PcG) proteins preserve transcriptionally silenced states through epigenetic marking of target genes in higher eukaryotes. Currently, at least two biochemically and functionally distinct polycomb repressive complexes (PRC) are recognized, PRC2 and PRC1, which contribute to establishment and maintenance of gene repression profiles (Figure 1a) [1-3]. As such, PcG function equips the cell with a transcriptional memory throughout development and differentiation. It is becoming increasingly clear that PcG-chromatin association is subject to dynamic regulation; PcG complex composition and chromatin association change throughout eukaryotic development [4,5]. In a constantly changing environment (for example, during differentiation), cells respond to a plethora of extracellular and intrinsic cues. Mechanistic insight in epigenetic regulation in response to signaling is essential to understand how cell fate, function and physiology are controlled. It is, however, still largely unknown how cells 'talk' to chromatin to facilitate appropriate cellular responses. Physiological adaptation of cells is initially mediated by mostly transient and reversible covalent post-translational modifications (PTMs) at specific amino acid residues including ubiquitylation, sumoylation, and phosphorylation (see Appendix). Through altered protein-protein interaction, subcellular localization, enzyme activity and protein stability, PTMs ultimately also affect gene expression. We review current established PTMs on PcG proteins and the effect they have on interaction and enzymatic activity. In addition, we extracted PcG-specific PTMs from published analyses and used these to predict upstream kinase pathways signaling to Polycomb. The final section presents a tentative integrated view on signaling to chromatin in the context of PcG PTM.

## Ubiquitylation: Polycomb-mediated ubiquitylation

Ubiquitylation plays a central role in PRC-mediated silencing. Histone 2A (H2A) is one of the most abundant ubiquitylated nuclear proteins, and H2AK119Ub1 is required for PcG-mediated gene repression [6]. Published data shows that the PRC1 protein ring finger protein 2 (RNF2, also known as RING1B and RING2) ubiquitylates H2A, as loss of RNF2 dramatically decreases global H2Aub levels and derepresses PcG-controlled genes. The RING domain of the really interesting new gene protein 1 (RING1, also known as RING1A) substitutes for that of RNF2 *in vitro *[7]. Consistently, H2AK119Ub1 is maintained in RING1 or RNF2 single null cells, but not in double knockout cells, supporting functionally redundant roles for these proteins in certain biological contexts [8]. RNF2-mediated H2A ubiquitylation is important in X chromosome inactivation [8,9]. The exact role of RNF2 and H2AK119Ub1 in X-inactivation is currently under debate, however, as recent studies suggest that H2AK119Ub1 may not be sufficient for X-inactivation and conversely, Xist-initiated silencing occurs in the absence of RNF2 and H2AK119Ub1 [10,11]. Additionally, histone variant H2A.Z may be a target for RNF2-mediated ubiquitylation, as knockdown of RNF2 reduces H2Aub and H2A.Zub levels *in vitro *[12].

Whereas in *Drosophila melanogaster *multiple E3 ligase complexes contribute to H2A ubiquitylation, the PRC1 protein RNF2 is currently considered the major E3 ligase for H2AK119 (Figure 1b) [13,14]. Although within the PcG core complex, containing RING1, RNF2, BMI1 and polyhomeotic homolog 2 (PHC2), most *in vitro *H2A-E3 ligase activity is attributed to RNF2 [8], other PcG RING-type E3 ligases control H2A-directed ubiquitylation and affect *HOX *gene silencing: BMI1 (Polycomb Group ring finger 4 (PCGF4)) and homologues PCGF2 (MEL18) and PCGF1 (NSPc1) enhance H2A ubiquitin (Ub) E3 ligase activity when complexed to RNF2 (Figure 1b) [15-18]. In addition, PcG ubiquitin E3 ligase activity is enhanced within the molecular context of an intact PRC1 complex: fully reconstituted complexes containing RNF2, RING1, BMI1 and chromobox homolog 8 (CBX8) show highest activity compared to RNF2 alone or subcomplete PRC1 complexes [15]. Crystal structure analyses of interacting RING domains of mammalian BMI1 and RNF2 reveals extensive contacts between the RNF2 and BMI1 RING domains: the N-terminal 'arm' of RNF2 embraces the BMI1 RING domain [7,19]. RING protein partnerships occur frequently in cell biology [20]; RING domain proteins function as adapters, bringing together E2 conjugases and their substrates (see Appendix) [21]. Based on structural analogy to a breast cancer type 1 susceptibility protein (BRCA1)-BRCA1-associated RING domain 1 (BARD1) complex, it was suggested that RNF2 contains an E2 binding site, whereas BMI1 is involved in substrate binding [19]. E2 ubiquitin conjugating enzymes (Ubc) UbcH5 subtypes a, b, c and UbcH6 promote H2A Ub conjugation, although these Ubcs do not bind the RING RNF2/BMI1 complex [7]. Which E2 conjugases contribute to H2A ubiquitylation *in vivo *is currently not known. A summary of PcG-related PTMs and their functional relevance is provided in Table 1.

**Table 1:** Post-translational modifications (PTMs) in polycomb group (PcG) biology

*PcG protein*	*PTM*	*Interacting protein*	*Biological effect*
RNF2 (and RING1)	Ub	H2A (K119), H2A.Z	Transcriptional repression, X inactivation
+ BMI1			Histone H2A ubiquitylation
+ phosphorylated MEL18			
+ PCGF1			
RNF2	Ub (mono, poly)	-	Protein stability,
BMI1	Ub	-	Protein stability
		CULLIN/SPOP3	X inactivation
RYBP	Ub	-	Histone H2A binding
PRC1	Ub	Geminin	Protein stability
CBX4	Sumo	CtBP	
		Dnmt3a	Abolishes repression
		HIPK2	DNA damage response
		SIP1	Relieves E-cadherin repression
		CBS	Inhibition CBS activity
PCGF2	Prevention of sumo	HSF2	Decreased interaction during mitoses
		RanGAP1	Increased interaction during mitosis
CBX4	Sumo	-	-
CtBP1	Sumo		
SUZ12	Sumo	PAISXβ	-
EZH2	Sumo	-	-
BMI1	Ph		Chromatin dissociation
CBX2	Ph	-	Nuclear localization
PCGF2/MEL18	Ph	PKC	Blocks dimerization
Esc (*Drosophila*)	Ph		Homodimerization, complex formation/stabilization
EED	Ph		Homodimerization
EZH2	Ph	-	Histone H3 binding
EZH1	Ph	ZAP70	Interaction
RNF2	Proteolytic cleavage	Caspases 3 and 9	Apoptosis
Ph (*Drosophila*)-PHC3	O-GlcNAc	*Sxc*/Ogt	Repression: unknown
RNF2	Ac	-	Unknown

Listed are published biochemical connections between PcG and associated proteins; see text for details on functional relationships.

Ph = phosphorylation; Su = sumoylation; Ub = ubiquitylation.

## Ubiquitylation of PcG proteins

The above relatively simple picture is complicated by additional levels of PTM. Both RNF2 and BMI1 are ubiquitin-conjugated proteins and differential autoubiquitylation of RNF2 is required for H2AK119Ub1 [7,22]. Polyubiquitylation of RNF2 requires Lys6, Lys27 and Lys48 linkage on the same ubiquitin molecule and RNF2 autoubiquitylation is promoted by Ubc5 *in vitro *[22]. Some of these K residues are involved in epigenetic silencing, as the ability of RNF2 to promote H2AK119Ub1 relates to the availability of UbK6 and UbK27, not UbK48 [22]. Although inhibition of Ub-dependent degradation with proteasome inhibitors increases RNF2 levels, autoubiquitylation mutant RNF2(I53S) proteins are still efficiently degraded, suggesting the involvement of other E3 ligase(s) and/or Ub site(s). BMI1 inhibits ubiquitylation of RNF2 and coexpression of RNF2 and BMI1 blocks its degradation in a RING domain-dependent manner [22]. Unlike RNF2, BMI1 lacks autoubiquitylation activity. However, like RNF2, it is stabilized by proteasome inhibition. As for RNF2, the identity of the ubiquitin E3 ligase responsible for proteasomal degradation of BMI1 is not known. Whereas RNF2K112ub appears dispensable for H2A E3 ligase activity [7], mutant RNF2 with an intact RING domain, however missing most ubiquitylation sites (K92-198R) still binds BMI1, but lacks E3 ligase activity [22]. Thus, seemingly at odds with each other: although RNF2 autoubiquitylation is required for H2A ubiquitylation, it is inhibited by BMI1, yet overall, BMI1 promotes RNF2 H2A-E3 ligase activity and blocks its proteolytic degradation [22]. A possible explanation for this discrepancy may involve number, length and linkage type of ubiquitin chains, aside from molecular context.

A candidate E3 ubiquitin ligase for BMI1 is the CULLIN3/Speckle-type POZ protein (SPOP) complex: *in vitro *and *in vivo *analyses confirmed that CULLIN3 and SPOP are required for BMI1 ubiquitylation in cells. As RNAi-mediated knockdown of CULLIN3 or SPOP does not affect BMI1 protein levels, CULLIN3/SPOP-mediated ubiquitylation of BMI1 most likely has no bearing on protein stability [23]. Interestingly, a human BMI1 polymorphism resulting in a C18Y substitution increases ubiquitylation and proteasomal degradation [24]. Whether or not this has any effect on human health is currently not clear.

The PcG protein RING1 and YY1 binding protein (RYBP) is monoubiquitylated by RING proteins, and binds H2AUb1, among other proteins, *in vitro*, through a zinc finger ubiquitin binding domain (UBD) of the Npl14 zinc finger (NZF) type. Although a UBD-NZF mutant still interacts with RING1 and RNF2, it prevents formation of Polycomb bodies in osteosarcoma cells, suggesting a dominant negative role in PcG recruitment [25]. Thus, RYBP may play a role in engagement of specific transcription factors, and hence direction of PcG complexes to specific target genes [17,26].

Combined, the above data shows that PRC proteins are engaged in numerous ubiquitin-dependent regulatory mechanisms, and that ubiquitylation is important for PcG-mediated silencing at multiple levels.

## Sumoylation: Polycomb-mediated sumoylation and Polycomb sumoylation

Although mechanistically not completely understood, one of the potential functional outcomes of PcG protein sumoylation is induction of transcriptional repression [27]. In support of a biologically relevant role in the context of transcriptional repression, sumoylation appears conserved throughout evolution. A genome-wide RNA interference screen in *Drosophila *cells identified proteins that, when absent, relieve small ubiquitin-like modifier (sumo)-dependent inactivation of the transcription factor Sp3 [28]. Among interactors identified were the PcG protein Sfmbt, the zinc finger protein MEP-1 and dMi-2, an ATP-dependent chromatin remodeler which shows genetic interaction with PcG [29]; all three proteins bind Sp3-sumo *in vitro *and all are recruited to promoters in a Sp3-sumoylation-dependent manner [28]. Additionally, in *Caenorhabditis elegans *a link was established between sumoylation and PcG proteins: the PcG-like protein SOP-2 interacts with UBC9 via its conserved sterile α motif/self association motif (SAM) domain [30]. SOP-2 sumoylation is required for *in vivo *localization to nuclear bodies and repression of *HOX *genes [30].

The identification of CBX4 as a sumo E3 ligase forged the first link between PcG function and sumoylation. C-terminus binding protein (CtBP; an interaction partner of RING1 and other PcG proteins [31]) is sumoylated [32,33]. CBX4 interacts with E2 Ubc9 and CtBP and sequesters both proteins to PcG bodies. Multiple biological CBX4 targets have been identified which begin to link PcG-mediated sumoylation to relevant biological processes [32,34-38]. Among these targets is Dnmt3a (*de novo *DNA methyltransferase). Despite its highly conserved nature among the PcG proteins CBX2, CBX6, CBX7 and CBX8, only the C-terminal COOH box of CBX4 interacts with Dnmt3a in a yeast two-hybrid setting. Dnmt3a is polysumoylated; sumoylation terminates the interaction of Dnmt3a with histone deacetylase (HDAC)1/2 and completely abolishes its repressive ability *in vitro*, suggesting a role for PTMs in dynamic epigenetic regulation of gene expression [35,36]. DNA damage-induced homeodomain interacting protein kinase 2 (HIPK2) phosphorylates and activates CBX4 E3 sumo activity, whereas CBX4-mediated sumoylation of HIPK2 in turn enhances its ability to repress genes in response to DNA damage (Figure 1c) [38]. This autoregulatory feedback loop is likely relevant in the context of cellular DNA damage responses. Sumoylation of SMAD interacting protein 1 (SIP1) interferes with CtBP interaction and relieves repression of E-cadherin, an important regulator of epithelial mesenchymal transition (EMT) during development and tumorigenesis [37]. Furthermore CBX4 targets cystathionine β-synthase (CBS), an enzyme involved in homocysteine to cysteine conversion [34]. Sumoylation of CBS inhibited its enzymatic activity [34]. As homocysteine to cysteine conversion is an important step in the synthesis of S-adenosylmethionine (SAM), a major methyl donor reagent for essential methylation reactions [39], this may have obvious implications for local and global epigenetic regulation.

In the context of PRC1 function, the PcG protein PCGF2, appears to oppose sumoylation. PCGF2 binds, both *in vitro *and *in vivo*, heat shock factor 2 (HSF2), which is predominantly sumoylated during mitosis [40]. PCGF2 dissociates from HSF2 during mitosis and, conversely, recombinant PCGF2 inhibits *in vitro *sumoylation of HSF2. Thus PCGF2 may act as an anti-sumo regulator [40]. PCGF2 also inhibits sumoylation of Ran GTPase activating protein (RanGAP)1, which is independent of its RING domain [41]. Intriguingly, in contrast to HSF2, the interaction between PCGF2 and RanGAP1 increases during mitosis, suggesting a sumo-dependent switch of interaction partners of PCGF2 [41].

PRC2 complex function is associated with sumoylation as well: suppressor of zeste 12 (SUZ12) and enhancer of zeste homolog 2 (EZH2) (Figure 1a) are both sumoylated; the E3 ligase for SUZ12 appears to be PAISXβ, not CBX4. The exact biological role of SUZ12 sumoylation is not clear, as wild type or non-sumoylatable SUZ12 3KR mutants both show similar H3K27me3 in a SUZ12^-/- ^background, and 3KR colocalization with EZH2 and embryonic ectoderm development (EED) is not affected [42]. An overview of experimentally confirmed site-specific sumoylation and ubiquitylation sites on PcG proteins is presented in Table 2. Although a total of 33 sumoylation sites are predicted on human PcG proteins, both their occurrence and biological relevance await validation *in vitro *and *in vivo *(Additional file 1).

**Table 2:** Site-specific polycomb sumoylation and ubiquitylation sites

Complex	*Drosophila*	Name	Alternative symbol	Accession no.	Total no. of amino acids	Modification site	Conservation in mouse	Refs
PRC2	Su(z)12	SUZ12		Q15022	739	K72 sumo	K74	[42]
						K73 sumo	K75	[42]
						K75 sumo	K77	[42]
PRC1	Pc	CBX4	HPC2	O00257	558	K492 sumo	K490	[38,99,100]
	Sce/dRING	Rnf2	RING1b	Q9CQJ4	336	K112 Ub	K112*	[7]
PhoRC	Pho	YY1		P25490	414	K288 sumo	K288	[101]

The table lists PTMs confirmed by mass spectrometric and/or mutational analysis.

Sumo = sumoylation site; Ub = ubiquitylation site.

In summary, sumoylation, like ubiquitylation, emerges as a PTM relevant for regulation of gene expression. In contrast to ubiquitylation, PcG-mediated sumoylation has not directly been linked to histone modifications yet. Instead, currently available data suggest a relevant role for sumoylation in dynamic interaction with non-PcG proteins in the context of cell physiology.

## Phosphorylation of PcG proteins

Although thus far no PcG proteins have been identified as kinases, phosphorylation is a common PTM on PcG proteins. Recent observations have shown that signaling, which triggers downstream phosphorylation events, affects subcellular localization, protein interactions within complexes, enzymatic activity and chromatin association of several PcG proteins and other factors. The first report of PcG protein phosphorylation suggested a meaningful regulatory role for PcG phosphorylation, as phosphorylated BMI1 dissociates from chromatin at late S-phase, when chromatin is assembled *de novo *[43]. Additionally, MEL18 and BMI1-like RING finger protein (MBLR; PCGF6) is predominantly phosphorylated in G2/M [44]. *In vitro *kinase assays suggested PCGF6 as a substrate for CDK7 [44]. Interestingly, Trithorax orthologs are also phosphorylated in a cell cycle-dependent manner [45], suggesting phosphorylation as a common mechanism to temporarily relocate chromatin-associated proteins. Phosphorylation may affect individual PcG proteins in other biologically relevant ways. CBX2/M33 phosphorylation affects nuclear localization: high mobility (unmodified) CBX2 resides in the cytoplasm in mouse livers, whereas low mobility isoforms localize to the nucleus [46]. Dimerization of PCGF2, is blocked in the presence of protein kinase C (PKC) [47]. Additionally, NSPc1/PCGF1 is functionally targeted by phosphorylation: a synthetic phosphomutant GAL4-DBD-NSPc1 no longer transcriptionally represses a GAL4-LUC reporter [48].

Phosphorylated *Drosophila *PRC2 protein *Extra sex combs *(Esc) is preferentially found in a 600 kDa complex with PcG protein *enhancer of zeste *(E(z)) and Esc phosphorylation is required for formation and stability of a larger Esc/E(z) complex containing PCL and RPD3 [49,50]. Both Esc and EED (its mammalian ortholog) are phosphorylated in a domain responsible for Esc/EED homodimerization [50]. Casein kinase (CK)1 and CK2 may be the responsible kinases, as they phosphorylate Esc and EED *in vitro *and promote Esc/EED homodimerization. In addition, phosphorylation appears to regulate EZH2, a PRC2 histone methyl transferase (HMT) which trimethylates lysine 27 on histone 3 (H3K27me3) [51]: AKT (protein kinase B)-induced EZH2 Ser21ph suppresses its methyltransferase activity by impeding EZH2 binding to histone 3 (Figure 1d) [52]. In addition, phosphorylation affects self-recruitment of EZH2 to its own mark, which may have bearing on maintenance of repression throughout cell divisions [53,54]. Additionally, the PcG EZH2 homologue EZH1 is phosphorylated: the tyrosine kinase p56^lck ^is required for the phospho-dependent association between EZH1 and zeta-associated protein 70 (ZAP-70) in activated T cells [55]; whether phosphorylated residues within EZH1 and ZAP-70 are directly involved in binding awaits experimental validation.

## Classification of PcG phosphorylation sites

**Figure 2:**
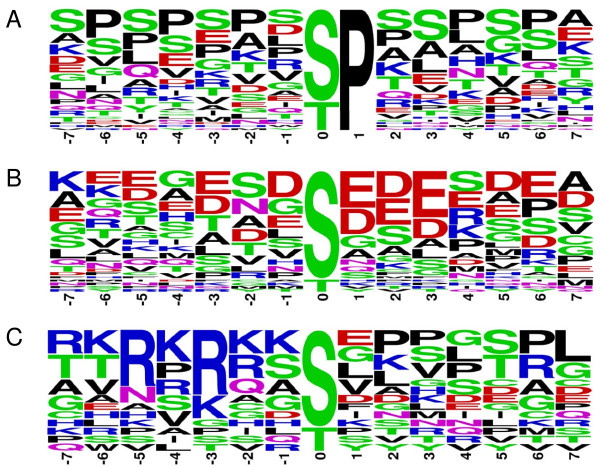
**Classification of S/T phosphorylation sites into general kinase recognition sequence categories**. At present, not all consensus phosphorylation motifs are known for all kinases. A number of general phosphosite classes have been annotated based on amino acid sequences surrounding S/T phosphorylation sites: proline-directed, acidic, basic and otherwise [97]. **(a-c) **Sequence logos for Polycomb Group (PcG) phosphorylation motifs of proline-directed (**(a)**, n = 42) acidic (**(b)**, n = 36) and basic (**(c)**, n = 15) categories where the phosphorylated residue (S/T) is centered were generated with Weblogo [98]. Only serine and threonine phosphorylation sites were taken into account when a full 15-mer sequence was available. To avoid sequence bias only non-overlapping human and mouse PcG phosphorylation sites were used. Centered 15-mer sequences were assigned to a motif class sequentially by following a binary decision tree as follows: P at +1 (Pro-directed), 5 or more E/D at +1 to +6 (acidic), R/K at -3 (basic), D/E at +1/+2 or +3 (acidic), 2 or more R/K at -6 to -1 (basic), otherwise. Colors correspond to the chemical properties of the amino acids: hydrophobic (black), basic (blue), acidic (red) and polar (green). **(a) **Although phosphosite numbers are low and thus no solid deductions are warranted, it is apparent for the proline-directed class (classified by a P in the +1 position), that prolines are not limited to +1, but are abundant in various - and + positions. **(b) **In the acidic motif, characterized by the presence of the amino acids glutamic acid (E) and aspartic acid (D) in the + residues, E/Ds are present in the - positions. **(c) **In basic motifs, arginine (R) is predominantly found in -3 positions, in addition to -5.

Most current published information on PcG phosphorylation neither unequivocally identifies responsible kinases nor targeted amino acid residues, and the majority of existing *in vitro *data will have to be confirmed and functionally tested *in vivo*. Over recent years several large-scale phosphoproteomic analyses have been published. We have extracted PcG-related data from these and our own studies; this reveals numerous PcG phosphorylation sites: to date 118 human (Table 3) and 31 mouse (Additional file 2) PcG phosphorylation sites have been published; so far 14 of these phosphorylation events are shared by both species (Table 4). Amino acids surrounding phosphorylation sites direct kinase binding and hence define kinase-substrate specificity. Classification of currently known PcG phosphorylation sites into predicted phosphosite categories shows that most sites group in the proline-directed or acidic class (Table 5; Figure 2a-c). Kinases that recognize/bind the proline-directed residues belong to the mitogen-activated protein (MAP) kinase (MAPK) family; acidic motifs are phosphorylated by CK2; kinases targeting basic motifs belong to the AKT/PKB and PKA types [56]. Consistent with our analysis, published PCGF2/6 (CK1/2) and EZH2 phosphorylation sites (AKT/PKB) classify in the acidic and basic target group, respectively.

**Table 3:** Human polycomb phosphorylation sites

Complex	*Drosophila*	Name	Alternative symbol	Accession no.	Total no. of amino acids	Phosphosite	Conservation in Mouse	Refs
PRC2	E(z)	EZH1		Q92800	747	-	-	-
		EZH2		Q15910	746	S21	S21	[52]
						T339	T339	[102]
						S362	S362	[103]
						S363	S363	[103]
						S366	S366	[102,103]
						T367	T367	[102,103]
						S380	S380	[104]
						T487	T487^a^	[102,103,105-110]
	Esc	EED		O75530	441	S25	S25	[102]^b^
						S34	S34	[102]
						S43	S43	[102]^b^
						S46	S46	[102]^b^
						T50	T50	[102]^b^
						T55	T55	[102]
						T57	T57	[102]
	Su(z)12	SUZ12		Q15022	739	S37	S37	[102]
						T131	T133	[111]
						S139	S141	[111]
						S382	S384	[103]
						T418	NC	[112]
						S541	S543	[102]
						S546	S548^a^	[102,103,105,107,110]
						S583	S585^a^	[103,107]
PRC1	Pc	CBX2	HPC1	Q14781	532	-	-	-
		CBX4	HPC2	O00257	558	Y205	Y207	[113]
						S347	S350	[102]^b^
						T349	T352	[102]^b^
						S413	S427	[110]
						T415	T412	[103]
						S430	S427	[107]
						T435	T432	[103]
						T495	NC	[38]
		CBX6		O95503	412	S107	S107	[103]
						S301	S302	[107]
						S303	S304	[107]
		CBX7		O95931	251	-	-	-
		CBX8	HPC3	Q9HC52	389	S110	S110	[102,108]^c^
						S130	NC	[102]
						T234	T207	[102]^b^
						S256	S229^a^	[102,103]
						S265	S238^a^	[102,103,108]^c^
						S311	S284^a^	^c^
						S332	S305	[102,107]
						S352	S325	[102]
						S354	S327	[102]^b^
	Ph	PHC1	HPH1/EDR1	P78364	1,004	Y32	Y32	[113]
						S195	S195	^c^
						S645	S653	^c^
						S653	S661	[114]
						S657	S665	[114]
						S664	S672	[114]^c^
						S669	S677	[114]^c^
						S786	S794	^c^
						S895	S903	^c^
						T918	T926	[103]
						T922	T930	[103]^c^
		PHC2	HPH2	Q8IXK0	858	S591	S583	[102]
						T619	T611	[102]
						S621	S613	[103]
						S701	S693	[104]
						S704	S696	[104]
						S733	S725	[115]^c^
						S745	S737	[105]
		PHC3	HPH3/EDR3	Q8NDX5	983	S229	S227	[115]
						S263	S261	[105,110]
						S315	S312	[102]^c^
						T609	T607^a^	[102,103,105,107,109,110]^c^
						T614	T612^a^	[102,107]^b^
						S616	S614^a^	[102,103,105,107,109,110]^c^
						S724	S722	[102]^b^
						S761	S759	[102]
						S762	S760	[102]
						S862	S860	^c^
	Sce/dRING	RING1	RING1A/RNF1	Q06587	406	S38	S38	[102,103,107]^c^
						S187	S187	[102,103]
						S188	S188	[102,103]
						S190	S190	[102,103]
						S229	S229	^c^
		RNF2	RING1B/RING2	Q99496	336	S41	S41	[69]
						S168	S168	[110]
	Psc	BMI1	PCGF4	P35226	326	S251	S249^a^	[110]
						S253	S251^a^	[110]
						S255	S253^a^	[110]
		PCGF2	RNF110/MEL18	P35227	344	Y197	Y197	[113]
						Y205	Y205	[113]
						Y206	Y206	[113]
						T344	gap	[102]
PhoRC	Pho	YY1		P25490	414	S118	S120^a^	[109]
						S184	S184	[104]
						S247	S247^a^	[102,103,105,108,109]
						Y251	Y251	[102]
						T348	T348	[102,116]
						T378	T378	[102,103]
Other	Psc	PCGF1	NSPc1	Q9BSM1	259	-	-	-
		PCGF6	MBLR/RNF134	Q9BYE7	350	S30^d^	S34	[44]
	Scm	SCMH1		Q96GD3	660	S649	S653	[102,114]
		SCML1		Q9UN30	208	S15	NA	[102]^b^
						S17	NA	[102]
						S117	NA	[108]
		SCML2		Q9UQR0	700	S255	NA	[102]^b^
						S256	NA	[102]
						S258	NA	[102]
						S267	NA	[102]
						S299	NA	[102]
						S300	NA	[102]
						T305	NA	[102]
						S495	NA	[102]^b^
						S499	NA	[102,103,105,106]
						T503	NA	[102]
						S511	NA	[102,103,105,106]
						S570	NA	[115]
						S576	NA	[102]
						T581	NA	[102]^b^
						S583	NA	[102]
						S590	NA	[102,103]
						S594	NA	[102,103]
						S606	NA	[102]
		RYBP		Q8N488	228	S127	S127	[102]
						S130	S130	[102]
						S203	S203	[102]^b^
						T215	T215	[102]^b^

^a ^Sites that were detected as phosphorylation sites in mouse cells as well; ^b ^these putative phosphorylation sites do not reach a sufficiently high probability based on prediction algorithms used; ^c ^marks unpublished results (HN, JD, JWV); ^d ^site was referred to as S32 in original article.

NA = not available; NC = non-conserved

**Table 4:** Summary of polycomb group (PcG) phosphorylation sites

Residue	Human	Mouse	Human + mouse non-overlapping	Human + mouse overlapping
Serine	87 (74%)	26 (83%)	101 (75%)	12 (86%)
Threonine	25 (20%)	5 (16%)	28 (21%)	2 (14%)
Tyrosine	6 (6%)	-	6 (4%)	-
Total	118 (100%)	31 (100%)	135 (100%)	14 (100%)

Classification of PcG phosphorylation sites based on common phosphoamino acids.

**Table 5:** Classification of polycomb group (PcG) S/T phosphorylation sites

Class	Human + mouse non-overlapping
Pro-directed	42 (37%)
Acidic	36 (32%)
Basic	15 (13%)
Other	20 (18%)
Total	113 (100%)

Classification of PcG phosphorylation sites in relation to amino acid context. Only putative Ser and Thr phosphorylation sites were taken into account when a full 15-mer sequence was available and a sufficiently high phosphorylation probability was predicted.

Although it is not known at present whether PcG phosphorylation directly affects chromatin association, their correlation (that is, signaling induced phosphorylation and chromatin dissociation) suggests cells use phosphorylation cascades to relay environment cues to chromatin, where reprogramming of gene activity includes epigenetic remodeling events [57]. Consistent with a functional link between MAPK signaling and PcG function, we recently showed that PRC1/chromatin association is disrupted downstream of MAPK activation [57]. Mitogen-activated protein kinase-activated protein kinase (MK3; MAPKAPK3, 3pK) associates with chromatin and occupies PcG target genes. Indeed, phosphopeptide analysis suggests that MK3 directly targets PcG proteins (HN and JWV, unpublished results). Although the involved PTMs have not been fully mapped, phosphorylation of PcG proteins affects specific protein interactions and, hence, PRC1 complex composition. Several observations underscore this hypothesis: immunofluorescence studies showed differential subnuclear localization of PHC1 compared to other PRC1 proteins downstream of signaling-induced phosphorylation [57]. Secondly, PHC1/2 proteins are not stably associated to the PRC1 core complex, but instead show increased heterotypical interaction (HN and JWV, unpublished results) [15,58]. Thirdly, PHC and CBX proteins show distinct chromatin enrichment/occupation profiles in response to physiological stimuli in chromatin immunoprecipitation studies; whereas CBX8 is mostly found on genes where H3K27me3 is also present, PHC1 enrichment profiles seem to be more global as are those for the PHC1 interacting kinase MK3 (HN and JWV, unpublished results).

**Figure 3:**
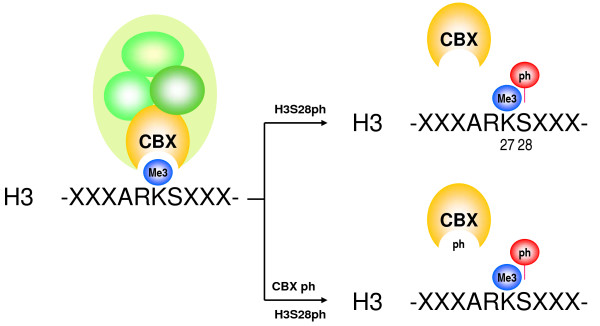
**Hypothetical mechanism for phosphorylation-induced dissociation of Polycomb Repressive Complex (PRC)1 from chromatin**. The chromodomain of chromobox homolog (CBX) proteins interacts with H3K27me3. Chromatin dissociation may be the result of ARKS motif methyl-phos switching, by phosphorylation of H3S28 (upper panel). Phosphorylation of conserved residue(s) within the chromodomain of CBX may contribute to chromatin dissociation (lower panel).

PcG/chromatin dissociation correlates with H3S28ph suggesting a functional analogy with H3S10ph and heterochromatin protein 1 (HP1) dissociation (see discussion below) [59]. Indeed MAPK signaling cascades target and phosphorylate H3 [60]. Regulatory analogy with HP1 may go further: DNA damage induces CK2-dependent HP1β T51 phosphorylation, which mobilizes HP1β and facilitates H2AX phosphorylation [61]. Dissociation involves disruption of hydrogen bonds required for HP1β chromodomain folding around H3K9me3. Although purely speculative at the moment, T51 is conserved in human and mouse CBX1 to CBX8 proteins (Ser in CBX2) and in HP1 and fruit fly Pc, these CBX residues may all function in release of methyl-binding interfaces (Figure 3). Of note, CK2 was recently identified as an interactor of RNF2 and CBX8 [62,63]. It is however currently unclear whether and how CK2 regulates PcG function.

In summary, it is clear that phosphorylation is a relevant part of PcG biology. Further characterization and functional validation of these phosphorylation sites will be pivotal for our understanding of the cellular conditions under which PcG phosphorylation takes place, the regulatory pathways involved and how cells use these pathways to modulate chromatin structure to allow biologically appropriate responses.

## Other PTMs in PcG biology

### *N*-Acetylglucosamine (GlcNAc)ylation

Recently, the *Drosophila *PcG gene *super sex combs *(*sxc*) was reported to encode the enzyme O-linked O-GlcNAc transferase (Ogt) [64]. PRC1 protein polyhomeotic (Ph) is GlcNAcylated by *sxc*/Ogt *in vivo*. PRC1-mediated repression is dependent on functional *sxc*/Ogt, possibly via GlcNAcylation of Ph [64]. Interestingly, the process appears conserved across species, as the mammalian ortholog PHC3 is O-GlcNAcylated as well [65]. Analogous to PcG, GlcNAcylation of the histone methyltransferase MLL5, a Trithorax-like protein, generally assumed to counteract PcG function, regulates methylation of H3K4 [66]. These findings begin to show that GlcNAcylation of chromatin factors, such as PcG proteins, is relevant for epigenetic regulation and provide important leads for future study.

### Proteolytic cleavage

Other than ubiquitin-26S proteasome-dependent protein degradation, as holds for PcG proteins RNF2 and BMI1 [22], proteolytic cleavage may also occur via activated caspases [67]. Caspase substrates include, among others, structural proteins, such as actin, vimentin and nuclear lamins and proteins involved in transcription and translation, such as nuclear factor (NF)κB and translation initiation factors [67]. Recently, RNF2 was identified as a direct substrate of caspases 3 and 9 in apoptotic cells [68]. The exact function of RNF2 cleavage during apoptosis remains illusive, but it is possibly a prerequisite for nuclear condensation and DNA fragmentation to occur. Whether PcG protein cleavage is relevant outside the context of apoptosis is currently unclear.

### Acetylation

Currently acetylation of only one PcG protein has been reported: RNF2 is acetylated at S2, which is accompanied by a N-terminal methionine excision [69]. The significance of this modification in relation to PcG function is not known.

## Signaling to chromatin: post-translational crosstalk to Polycomb

It is evident that PcG proteins are subject to numerous different PTMs (among others, ubiquitylation, sumoylation, phosphorylation and GlcNAcylation) and, besides, harbor intrinsic enzymatic modifying activities themselves (HMT, ubiquitin and sumo E3 ligase, O-GlcNAc transferase). How these PTMs relate to each other is currently far from clear, however, several studies begin to unveil an intricate interplay between different PTMs, their effect at the molecular level and their biological relevance (Figure 1). Complex formation between PCGFs and RNF2 regulates H2A ubiquitin E3 ligase activity [16]. PCGF2 phosphorylation (Additional file 2) is required for substrate recognition in a relevant chromatin context, as dephosphorylated RNF2/PCGF2 complexes no longer efficiently ubiquitylate nucleosomes [16]. Similar regulation likely holds true for its close relatives BMI1 and PCGF1. Phosphorylated EZH2 has reduced methyltransferase activity toward histone 3, as a result of reduced substrate binding [52]. Phosphorylation and sumoylation-mediated autoregulatory feedback between CBX4 and HIPK2 has obvious relevance for DNA damage responses [38]. Recently the PRC1 complex was identified as the E3 ubiquitin ligase for geminin, an inhibitor of replication licensing factor Cdt1. PHC1 (Rae28) deficiency in mice impairs ubiquitin/S26-proteasome-mediated degradation of geminin and has direct consequences for cell cycle progression in the haematopoietic lineage [70].

**Figure 4:**
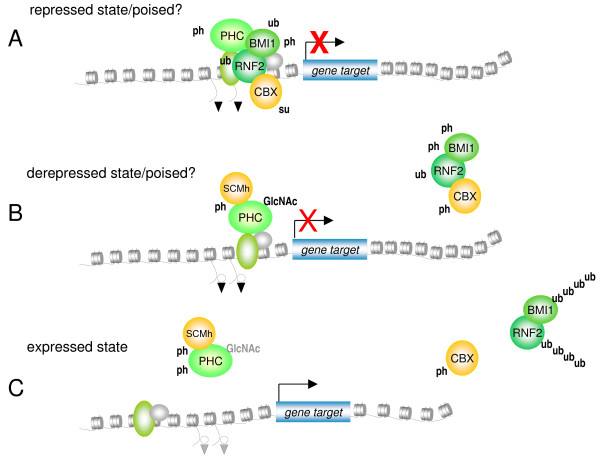
**Integrated hypothetical model for post-translational modification (PTM)-dependent regulation of Polycomb Repressive Complex (PRC)1-mediated repression**. Three independent chromatin states in the context of PRC1 function are recognized: **(a) **the repressed state, which requires ubiquitylation and sumoylation of PRC1 compounds. Detectable baseline phosphorylation may indicate a prerequisite for PRC function and/or differences in PRC activity at local targets throughout the genome. Signaling to chromatin alters PTM states and chromatin association, and eventually releases PRC silencing **(b,c)**. Observations from our and other groups suggest differential regulation of polyhomeotic homolog (PHC) proteins versus RNF2, BMI1 and chromobox homolog (CBX) proteins; this may involve *N*-acetylglucosamine (GlcNAc) modification in mammals as well. **(c) **Whether or not full expression of a Polycomb Group (PcG) target gene requires complete removal or relocation of PHC is currently not known. Likewise, whether ubiquitin-mediated proteolysis of BMI1 and RNF2 is needed to release gene repression is purely speculative. ph = phosphorylation; su = sumoylation; ub = ubiquitylation. Black triangles = H3K27me3; open circles = H3S28ph; unmarked circular/oval structures represent general transcription factors and/or unknown proteins.

In keeping with differential chromatin association of Ph as compared to other PRC1 proteins, GlcNAcylated Ph does not copurify with other PRC1 components in larval extracts, whereas it does in embryonic nuclear extracts [64]. Phosphorylation and GlcNAcylation mostly affect the same amino acids (S/T), hence an intricate interplay between these modifications may be predicted, either via competitive occupancy at the same site or alternative occupancy at adjacent sites [71]. Taken together these observations support differential regulation within the PRC1 complex upon activation of signaling cascades (Figure 4), implicitly suggesting differential roles for and/or regulation of PHC proteins and other members of the PRC1 complex in response to cellular signaling.

## Integration of signaling at the chromatin level

Signaling-induced PTMs clearly affect non-histone and histone proteins concurrently, suggesting that signaling-induced phosphorylation evokes an integrated response at the level of chromatin-associated proteins and nucleosomes. Histone H3Ser phosphorylation occurs downstream of signaling induced by arsenite (Akt1, extracellular signal-related kinase (ERK2) and RSK2), ultraviolet B (ERK, p38 MAPK, c-Jun N-terminal kinase (JNK1) and MSK1), TPA (MSK2 and MSK1) and anisomycin (MSK2 and Mitogen and stress-activated protein kinase-1 (MSK1)) [72-76]. Even though MSK1 phosphorylates histone H3S10 and S28 within the same peptide *in vitro*, this apparently does not happen *in vivo*, where subnuclear localization of histone H3S10ph and H3S28ph are distinct [77,78]. The spatial distribution of these H3 PTMs is likely controlled by context-dependent kinase recruitment (by, for example, HP1 or PcG complexes).

Although the correlation between PcG phosphorylation and chromatin dissociation is clear, both cell cycle dependent and cell cycle independent underlying molecular mechanisms remain largely obscure [43,44,57]. Comparison of PcG modules to a related histone binding protein family, HP1 (associated with constitutive heterochromatin and regions of facultative heterochromatin) may begin to reveal molecular mechanisms [79]. HP1 and PcG CBX proteins share a chromodomain, which binds specific histone 3 trimethyl marks. Both chromodomain protein classes are released from chromatin during mitosis. HP1 dissociates from chromatin in M-phase, despite unchanged H3K9me3 levels. Aurora kinase B-induced phosphorylation of H3S10 peptides adjacent to the K9me3 mark strongly reduces HP1 binding *in vitro *and releases HP1 from chromatin *in vivo *[80]. H3S10 phosphorylation was put forward as part of a 'binary switch' mechanism of the 'methyl/phos' type: phosphorylation adjacent to a methyl mark leads to induced loss of methyl-based binding of a factor or complex [59,81]. Dynamic 'methyl/phos' switching modules also provide a tentative molecular basis for heritable transcriptional memories. Such a switching mechanism may not be limited to the K9/S10 region of H3, but may be more common: both K9 and K27 in histone 3 reside in nearly identical amino acid contexts (ARKS); hence 'methyl/phos' switching may control CBX binding to H3 as well (Figure 3). Indeed, H3S28 is also phosphorylated in M-phase [82,83]. Relevantly, we reported a close correlation between signaling-induced PRC1 dissociation and H3S28ph, whereas this was not seen in relation to H3S10ph [57], suggesting similar molecular strategies between related proteins. Hence, the larger family of MSK, ERK and RSK kinases integrate regulation of gene expression at several functional levels by targeting transcription factors, chromatin binding complexes and nucleosomal components.

Although clearly numerous PTMs affect PcG biology, molecular mechanisms in signaling to chromatin, downstream modulation of epigenetic marking and the establishment and transfer of heritable epigenetic states remain largely elusive. Specific consensus/motif based kinase-substrate interactions most likely define and direct signaling-induced remodeling and/or other epigenetically relevant events at the chromatin level. In contrast, context-dependent variation in sumoylation sites is less well defined and the role of select consensus motifs in ubiquitylation is largely unknown. Although speculative, this may suggest that once signaling is triggered, a hierarchical sequence of PTMs is initiated, the target specificity (that is, networks, pathways, complexes) of which is defined by phosphorylation events, whereas downstream effects (among others, altered protein interactions, activity or stability) are 'merely' consequential, and controlled by numerous other PTMs. Indeed ubiquitylation, sumoylation, GlyNAcylation and phosphorylation are probably functionally linked in PcG biology, as important cross talk between these PTMs exists in many ways in other systems [84]. In this context important open issues are for instance how exactly PcG PTMs functionally relate to each other, that is, whether PTMs act separately, processively or combinatorially. Signaling-induced PTMs are generally reversible, proteolytic cleavage excluded, hence many chromatin-associated epigenetic regulators are presumably rapidly converted to their initial post-translational status upon signal recession. However, at the receiving end, stable, heritable covalent histone modifications appear somehow exempt from such regulation. Notions such as these should provide important basis for future hypothesis-driven research.

## Conclusion

It is evident that chromatin-associated protein complexes, like PcG proteins, are targets for cell signaling. These signaling events lead to PTMs that may affect chromatin binding, complex composition and catalytic activity. We and others have found that multiple kinases target PcG proteins. In addition, PcG proteins are subject to ubiquitylation, sumoylation and additional PTMs. Studies reviewed in this manuscript have only just begun to unravel the complexity and multiple layers of regulation of PcG function. PcG-mediated transcriptional silencing already appears a much more complex process than the antiquated view that PRCs physically obstruct transcription factor binding to DNA, and ongoing studies refine positioning of PcG function in the proper cellular context. Current conservative estimates predict the existence of anywhere between 60 to 100 or more mammalian PcG(-related) proteins, each likely with multiple phosphorylation and/or ubiquitylation, sumoylation and potentially numerous other PTM sites, including acetylation and methylation [85,86]. In stark contrast with this, at the moment only two PcG phosphospecific antisera exist [44,52]. Clearly there is a need for additional experimental tools and approaches.

Ultimately PTMs are aimed at concerted regulation of a number of chromatin-based processes in which PcG proteins play a role, including dynamic transcriptional regulation, long-term silencing, DNA replication and DNA damage responses, to ensure proper regulation of cell fate and survival. Increased insight into mechanisms employed by cells to target chromatin and chromatin-associated factors, including PcG, and their physiological consequences at the chromatin level will be important for further development and application of epigenetic strategies in for instance regenerative medicine. As many of the processes targeting and involving PcG function are etiologically linked to disease (for example, overexpression in cancer, bypass of replicative senescence [62,87-89]), a better fundamental understanding of gene-environment interactions at the molecular level will eventually contribute to the development of therapeutic and preventive strategies relevant for Western world-type diseases, including obesity, diabetes and cancer.

## Competing interests

The authors declare that they have no competing interests.

## Authors' contributions

HN wrote the initial draft and JD and JWV edited the manuscript. All authors have read and approved the final manuscript.

## Appendix: Most common PTMs in polycomb

### Ubiquitylation

Besides triggering protein degradation ubiquitylation fulfills many non-proteolytic roles, such as in regulating DNA repair, transcription and signal transduction [90]. Ubiquitin is a small ubiquitous 76 amino acid polypeptide which is covalently bound as a monomer (monoubiquitin) to proteins or as a Ub polymer (polyubiquitin). In polyubiquitin, chains are linked by conjugation to one of seven lysines of a pre-existing ubiquityl moiety, for example, Lys48 or Lys63. Polyubiquitylation via Lys48 linkage is mostly implicated in 26S proteasome-mediated protein degradation. Covalent linkage of ubiquitin to a substrate requires sequential action of activating (E1), conjugating (E2) and ligating (E3) enzymes [91]: E1 activates ubiquitin in an ATP-dependent manner, which is then conjugated to the E2, that, assisted by an E3 ligase, transfers ubiquitin to a lysine residue onto the substrate protein [90]. The diversity and combination of these enzymes ultimately determines substrate specificity and biological responses. Two classes of E3 ligases are recognized: the HECT domain E3s and the RING domain E3s; RING-type E3 ligases are thought to function as bridging factors between the E2 and substrate [91]. Ub protein substrates are deubiquitylated by deubiquitylases (DUBs). The large number of different E2 and E3 enzymes and DUBs identified to date and their involvement in a great variety of processes underscores the complexity of regulation by ubiquitylation [91].

### Sumoylation

Sumo proteins are approximately 10 kDa in size [92]. Like ubiquitylation, sumoylation involves three distinct enzymatic activities: an E1 activating enzyme (AOS1/UBA2 heterodimer) an E2 conjugating enzyme (UBC9) and an E3 ligase. Currently three classes of sumo E3 ligases have been distinguished: SP-RING motif E3 ligases, such as protein inhibitor of activated STAT (PIAS) proteins, a second class containing RanBP2, and a third class with no apparent homology to either other class comprising CBX4 (HPC2). In contrast to ubiquitylation, sumoylation takes place at sumo acceptor sites: ΨKXE (Ψ and X represent hydrophobic and any amino acids).

### Phosphorylation

Phosphorylation is probably the most widespread and best studied PTM in cellular signaling. Protein kinases catalyze the transfer of γ-phosphate from ATP to a substrate protein, thereby generating ADP [93]. Phosphorylation is reversible: phosphatases remove the attached phosphate moiety. An estimated 30% of all proteins are phosphorylated on at least one residue. Phosphate is most commonly linked to Ser (S), Thr (T) or Tyr (Y)), but in addition occurs on His (H), Lys (K), Arg (R), Asp/Glu (D/E), and Cys (C) [84].

## Supplementary Material

Additional file 1 **Prediction of human polycomb sumoylation sites**. A table containing predicted sumoylation sites on human PcG proteins. Click here for file

Additional file 2 **Mouse polycomb phosphorylation sites**. A table containing published mouse polycomb phosphorylation sites. Click here for file
