# Supplementary material for: Talking to chromatin: post-translational modulation of polycomb group function
Source: Epigenetics Chromatin. 2009 Sep 1;2:10. doi: 10.1186/1756-8935-2-10 (PMC2745409; doi:10.1186/1756-8935-2-10)
Supplement: Additional file 1 — Prediction of human polycomb sumoylation sites. A table containing predicted sumoylation sites on human PcG proteins. [file 1756-8935-2-10-S1.pdf]

Supplemental Table 1: Prediction of human Polycomb Sumoylation Sites

Complex	Drosophila	Name	Accession Number	Total nr AA	Position	Peptide	Type
PRC2	E(z)	EZH1	Q92800	747	20	RKVKSEY	Ψ-K-X-E
					140	DEVKEED	Ψ-K-X-E
					327	KEIKIEP	Ψ-K-X-E
		EZH2	Q15910	746	20	KRVKSEY	Ψ-K-X-E
	Esc	EED	O75530	441	19	PAAKKQK	Non-consensus
	Su(z)12	SUZ12	Q15022	739	75#	KKPKMEH	Ψ-K-X-E
					145	EKMKGEQ	Ψ-K-X-E
					735	KQSKKQK	Non-consensus
PRC1	Pc	CBX2	Q14781	532	153	LVAKPEL	Ψ-K-X-E
					410	DTSKSEK	Non-consensus
		CBX4	O00257	558	492#	LQVKPET	Ψ-K-X-E
		CBX6	O95503	412	-		
		CBX7	O95931	251	-		
		CBX8	Q9HC52	389	-		
	Ph	PHC1	P78364	1004	799	NLLKCEY	Ψ-K-X-E
					976	LLLKEEH	Ψ-K-X-E
		PHC2	Q8IXK0	858	641	LKLKCEL	Ψ-K-X-E
		PHC3	Q8NDX5	983	784	ELLKCEF	Ψ-K-X-E
					981	NSLKES*	Non-consensus
	Sce/dRing	RING1	Q06587	406	-		
		RNF2	Q99496	336	336	KEHK***	Non-consensus
	Psc	BMI1	P35226	326	88	GLFKNEM	Ψ-K-X-E
		PCGF2	P35227	344	88	GLFKDEM	Ψ-K-X-E
PhoRC	Pho	YY1	P25490	414	-		
Other	Psc	PCGF1	Q9BSM1	247	-		
		PCGF6	Q9BYE7	350	16	GAAKTEG	Ψ-K-X-E
	Scm	SCMH1	Q96GD3	660	63	ISMKLEA	Ψ-K-X-E
					172	MGMKLEA	Ψ-K-X-E
		SCML1	Q9UN30	208	-		
		SCML2	Q9UQR0	700	13	MDVKKEN	Ψ-K-X-E
					68	VGMKLEA	Ψ-K-X-E
					161	TLFKKEP	Ψ-K-X-E
					177	VGMKLEA	Ψ-K-X-E
					252	NIAKTES	Ψ-K-X-E
					487	QSAKEDV	Non-consensus
					536	AIPKEEN	Ψ-K-X-E
		RYBP	Q8N488	228	77	PPPKKEK	Ψ-K-X-E
					80	KKEKKEK	Non-consensus
					83	KKEKVEK	Non-consensus
					86	KVEKQDK	Non-consensus

Predictions were made by using SUMOsp 2.0, a site-specific predictor of sumoylation sites [1].
#: experimentally verified sumoylation sites (see Table 2 in main text).

References

1. Ren J, Gao X, Jin C, Zhu M, Wang X, Shaw A, Wen L, Yao X, Xue Y: **Systematic study of protein sumoylation: Development of a site-specific predictor of SUMOsp 2.0.** *Proteomics* 2009.
