# Supplementary material for: Talking to chromatin: post-translational modulation of polycomb group function
Source: Epigenetics Chromatin. 2009 Sep 1;2:10. doi: 10.1186/1756-8935-2-10 (PMC2745409; doi:10.1186/1756-8935-2-10)
Supplement: Additional file 2 — Mouse polycomb phosphorylation sites. A table containing published mouse polycomb phosphorylation sites. [file 1756-8935-2-10-S2.pdf]

Supplemental Table 2: Mouse Polycomb Phosphorylation Sites

Complex	Drosophila	Name	Alternative symbol	Accession number	Total nr AA	Phosphosite	Conservation in Humans	Ref
PRC2	E(z)	EZH1		P70351	747	-		
		EZH2		Q61188	746	T487	T487*	[1]
	Esc	EED		Q921E6	441	-		
	Su(z)12	SUZ12		Q80U70	741	S548	S546*	[1]
						S585	S583*	[1]
PRC1	Pc	CBX2	m33	P30658	519	-		
		CBX4	mPc2	O55187	551	-		
		CBX6		Q9DBY5	414	-		
		CBX7		Q8VDS3	158	-		
		CBX8	mPc3	Q9QXV1	362	S229	S256*	[2]
						S238	S265*	[2]
						S284	S311*	[2]
						T286	NC	[2] [3]
	Ph	PHC1	mPh1/Edr1/Rae28	Q64028	1012	-		
		PHC2	mPh2/Edr2	Q9QWH1	850	-		
		PHC3	mPh3	Q8CHP6	981	S231	NC	[2]
						S269	S272	[3]
						T607	T609*	[2] [3]
						S612	S614*	[2] [3]
						S614	S616*	[2] [3]
	Sce/dRing	RING1	Ring1A	O35730	406	-		
		RNF2	Ring1b	Q9CQJ4	336	-		
	Psc	BMI1		P25916	324	S110	S110	#
						S249	S251*	[3], #
						S251	S253*	[3]
						S253	S255*	[3]
						S314	S316	#
		PCGF2	Mel18	P23798	342	S110	S110	[4]
						S132	S132	[4]
						S254	S254	[4]
						S258	S258	[4]
						S260	S260	[4]
						S265	S265	[4]
						S278	S278	[4]
						T281	T281	[4]
						S286	S286	[4]
						S299	S299	[4]
PhoRC	Pho	YY1		Q00899	414	S120	S118*	[1]
						S247	S247*	[1] [3]
Other	Psc	PCGF1	Nspc1	Q8R023	259	-		
		PCGF6	Mblr/Rnf134	Q99NA9	353	S118	S115	[1]
	Scm	SCMH1		Q8K214	706	-		
		Rybp		Q8CCI5	228	S227	S227	[1]

(*): sites that were detected as phosphorylation sites in human cells as well; AA: Amino Acid; NC: non-conserved; # unpublished results (HN,JD,JWV)

References

1. Li H, Xing X, Ding G, Li Q, Wang C, Xie L, Zeng R, Li Y: **SysPTM - a systematic resource for proteomic research of post-translational modifications.** *Mol Cell Proteomics* 2009.
2. Villen J, Beausoleil SA, Gerber SA, Gygi SP: **Large-scale phosphorylation analysis of mouse liver.** *Proc Natl Acad Sci U S A* 2007, **104:**1488-1493.
3. Zanivan S, Gnad F, Wickstrom SA, Geiger T, Macek B, Cox J, Fassler R, Mann M: **Solid tumor proteome and phosphoproteome analysis by high resolution mass spectrometry.** *J Proteome Res* 2008, **7:**5314-5326.
4. Elderkin S, Maertens GN, Endoh M, Mallery DL, Morrice N, Koseki H, Peters G, Brockdorff N, Hiom K: **A phosphorylated form of Mel-18 targets the Ring1B histone H2A ubiquitin ligase to chromatin.** *Mol Cell* 2007, **28:**107-120.
